# PHARMACEUTICALS: New Insights into Thalidomide

**Published:** 2009-02

**Authors:** Valerie J. Brown

Thalidomide, a drug banned in some countries for nearly 50 years because it causes malformations in embryonic limb tissue, may be on the verge of a dramatic, if carefully circumscribed, reinstatement for use against a range of diseases. Its return to the fold may be aided by a new study in the 1 December 2008 issue of *Molecular Pharmaceutics* that further elucidates the mechanism by which thalidomide caused teratogenic effects. This understanding, in turn, may help prevent similar damage in new applications for the drug and its analogues.

First made in Germany in the 1950s, thalidomide was marketed as a sedative and anti-emetic. Rodent tests indicated thalidomide had low toxicity. It is now known, however, that most rats and mice are insensitive to the teratogenic effects of thalidomide, whereas humans, chickens, and rabbits are highly sensitive. By 1961 about 10,000 babies with severe limb malformations had been born to women who took the drug while pregnant, and thalidomide was removed from the market.

Interest in thalidomide continued, however. Today, cancer researchers are very interested in thalidomide’s cancer-curbing potential, which has been primarily associated with the inhibition of angiogenesis, the growth of new blood vessels recruited by the tumor. The U.S. Food and Drug Administration approved the drug in 1998 for treating the skin lesions associated with leprosy and in 2007 for treating newly diagnosed multiple myeloma, as long as strict protocols to prevent pregnancy are followed in female patients of child-bearing age. A number of small-scale studies have suggested the drug would be effective against other inflammatory diseases including rheumatoid arthritis, inflammatory bowel disease, and mucous membrane ulcers in HIV-positive patients.

The *Molecular Pharmaceutics* report, by Jürgen Knobloch and colleagues at Heinrich-Heine-University in Düsseldorf, Germany, builds on earlier work by the team showing that thalidomide triggers massive apoptosis in embryonic tissue responsible for outgrowth of the limbs. This cell death results from production of reactive oxygen species (ROS), leading to overwhelming oxidative stress in those cells.

In the current study, Knobloch and colleagues dosed chicken, human, and mouse embryonic fibroblasts with thalidomide and, after an incubation period, measured the cells’ levels of superoxide, a powerful free radical. They also measured levels of glutathione, a major antioxidant inside cells that affords protection against superoxide and other ROS.

As expected, the human and chicken fibroblasts were unable to neutralize the ROS and ward off cell death. Because thalidomide caused so many cells to die, the embryonic limb buds failed to develop properly. The mouse fibroblasts were able to produce enough antioxidants to protect the developing tissue, but the researchers also found that if they depleted glutathione in these cells, the effects were similar to those in the thalidomide-sensitive species.

Many scientists now speculate that thalidomide’s therapeutic promise against various diseases ultimately will be fulfilled not by the drug itself, but by analogues that do not share its undesirable effects. “It is clear that prolonged exposure results in unacceptable neurotoxicity,” says W. Douglas Figg, head of the Molecular Pharmacology Section and the Clinical Pharmacology Program of the National Cancer Institute’s Medical Oncology Branch. “There are still unanswered questions, especially [regarding] the relationship between birth defects and anticancer effects.”

Knobloch considers answering those questions “a prerequisite for developing efficient thalidomide derivatives for cancer and inflammation-based diseases.” His team is now investigating thalidomide’s anti-inflammatory effect in hopes of clarifying the associated mechanism and eventually finding treatments for inflammatory lung diseases.

